# Validation of an epilepsy management smartphone application in Pakistan

**DOI:** 10.1186/s42494-021-00075-9

**Published:** 2022-02-07

**Authors:** Victor Patterson, Awais Bashir Larik, Mohamed Zaman Soomro, Steve Coates

**Affiliations:** 1 Department of Medicine, University of Khartoum, Khartoum, Sudan; 2 Department of Neurology, Peoples University, Nawabshah, 67450 Pakistan; 3 Epilepsy Medicare Foundation, Nawabshah, Pakistan; 4 TeleEEG, Whitley Bay, NE25 8EE UK

## Abstract

**Background:**

There is no single way to improve epilepsy care in low- and middle-income countries (LMICs). An epilepsy management aid application (app) has been described, which enables a non-physician health worker (NPHW) to communicate with an epilepsy specialist using a smartphone. In this study, we aimed to assess the validity and quality of this care system in building connections between NPHWs and specialists in Pakistan and the UK.

**Methods:**

A NPHW in Pakistan used the app on a series of referrals and sent the app-generated summary by email to a neurologist in the UK, who replied and suggested possible management. Patients were later seen in a face-to-face (FF) manner by the UK neurologist and a local neurologist, and diagnostic accuracy and quality parameters were assessed.

**Results:**

Over 10 months, 59 patients were recruited and 33 of them were available for FF assessment. The misdiagnosis rate of the app was 6% (2 cases). Treatment advice provided by the app was judged appropriate in 32 patients (97%). In addition, 46% of the referrals were completed within 2 h and 85% within 24 h.

**Conclusions:**

Consistent with an earlier study, this system is a safe method to provide care for patients who cannot access neurological services in person. In addition, it has advantage of timeliness compared to FF assessment and requires less specialist time, both of which are especially important during the coronavirus disease 2019 pandemic. This system can be generalised easily, depending on the willingness of referrers and specialists to use it.

## Background

Epilepsy is a significant public health problem in low- and middle-income countries, and has recently been extensively summarised [1]. Patients with epilepsy are often left untreated or poorly-treated in these countries, resulting in excess mortality [2, 3], injury such as burns, and considerable stigma affecting both the persons with epilepsy (PWEs) and their families [4].

The problem is due in large part to the lack of access to doctors [5], which is because there are few doctors in where PWEs live, or the doctors do not feel competent managing epilepsy which is perceived by them as complicated. One of the suggestions to improve this state is to train non-physician health workers (NPHWs) to diagnose and manage epilepsy, as suggested by the World Health Assembly [6]. Therefore, support should be given to them. In earlier studies, we have described a smartphone application (app), Epilepsy Diagnosis Aid, that enables NPHWs to diagnose an epileptic episode [7]. This app has been validated in different populations in India and Nepal [8], is easy to use by computer-naive NPHWs [9], and when used by them has a diagnostic certainty similar to non-specialist doctors [10].

An app for episode diagnosis only is, however, limited in what it can do. Although the diagnosis of an episode as epileptic or otherwise is an important step, there are other steps important for epilepsy management. Therefore, there is a need for an effective and extensive tool for NPHWs beyond episode diagnosis. This led us to develop a more comprehensive app, the Epilepsy Management Aid, which incorporates the sorts of questions which epilepsy specialists ask in diagnosing and managing epilepsy (Table 1). The development and structure of the Epilepsy Management Aid app (currently available for Apple and Android devices) has been described in detail previously [11]. In this study, we describe its use, with a specific focus on its safety, by an NPHW in Pakistan.

**Table 1:** Questions asked by epilepsy specialists for epilepsy management, which are incorporated in the Epilepsy Management Aid app

Temporal characteristics of episodes	
Are the episodes epileptic?	
What is their frequency?	
Are they provoked?	
Are they acute symptomatic seizures?	
What other seizures are present?	
What is the epilepsy type?	
What investigations have been done?	
What are the current medications?	
Are there drug allergies?	
What is the best treatment?	

## Methods

### Features of the epilepsy management aid app

The management app records answers to the questions in Table 1 and also has other functions as shown in Table 2.

**Table 2:** Functionality of the Epilepsy Management Aid app

Record answers to questions shown in Table 1	
Generate a summary	
Send the summary to another doctor by email or messaging	
Display records on the phone or tablet	
Upload records to a secure server	
Download records as a .csv file	

The app records answers to the questions in Table 1 as described in detail in the previous paper [11]. It then generates a summary which can be sent by text message or email to a more experienced doctor who can reply with suggestions on management.

For untreated epilepsy, the app suggests management based on sex, age and epilepsy type, but for treated epilepsy, referral to a doctor is suggested; this could either be a face-to-face (FF) consultation with a local doctor or a telemedical consultation to a remote doctor using the app-generated summary as the patient record.

### Location

Nawabshah is a city in Sindh Province of Pakistan. The Epilepsy Medicare Foundation of Nawabshah is a non-governmental organisation dedicated to supporting epilepsy patients living around Nawabshah, where there is a population of ~ 300,000. The effects of epilepsy in this population have been described previously [12]. One of the authors, MZS, is President of the Epilepsy Medicare Foundation and a trained neurophysiology technician. Apart from providing clinics for PWE and their families, he provides a range of clinical neurophysiological services to support the work of the organisation. The present study arose from the involvement of one of the authors, SC, in providing a tele-EEG service to this clinic. MZS speaks good English.

### Patients

We included the first 59 consecutive patients who were seen between October 2018 and August 2019. They were mostly poor patients who had been referred directly to the Epilepsy Medicare Foundation. The method used was as follows: MZS saw the patients, completed the app, and sent the app-generated summary by email to VP, who replied either suggesting management or asking for further information if clarification was needed, which sometimes resulted in a change of the diagnosis or management from that made by the app. We analysed the time to first reply and, if further information was requested, the time to final reply as well as the changes in diagnosis or management. These patients also underwent an EEG recording performed locally and the EEG was interpreted through the UK-based charity TeleEEG (www.teleeeg.org). Reports were not available when the initial reply was made. Comparison of the app results and EEGs will be the subject of a separate paper.

### FF assessment

All patients were offered a FF assessment after the app use. Patients were seen by two neurologists, ABL and VP. The consultation was carried out in the Sindhi language by ABL and the information listed in Table 3 was recorded. When seeing the patients, the neurologists were blind to both the app results or the diagnosis and management provided by the specialist. This FF encounter was taken as the “gold standard”.

**Table 3:** Information acquired during the FF assessment

Number of episodes in last 3 months	
Months since last episode	
Current treatment	
Episode diagnosis	
Other seizure types	
Epilepsy type	
Better/worse/same	

## Results

### Patients

Fifty-nine patients were recruited between October 2018 and August 2019, with a mean age of 20 years (Age range, 7 to 60 years; median, 18 years), and 59% of them were male.

### Timeliness of response

The initial reply to the email referral was within 2 h in 61% and within 24 h in 98% of the 59 patients (mean 4.7 h, median 1.2 h). Twenty-three patients (39%) were required to give more information via a further exchange of emails. Taking these cases into account, 46% of referrals were *completed* within 2 h and 85% within 24 h (mean 14.5 h, median 2.2 h).

### Comparison with FF assessment

Thirty-three of the 59 patients (56%) were available for FF assessment. This proportion was lower than anticipated, but may be justified by the fact that many patients, particularly those from poorer countries, have more to do to make a living than attending medical appointments which are for the benefit of doctors rather than themselves. The follow-up interval between first referral and the FF appointment ranged 1–10 months.

### Assessment of episodes

The diagnosis of one patient on FF examination could not be agreed between the two neurologists, so the patient was excluded from the analysis. The two neurologists agreed that 30 patients had epilepsy, of whom 29 were diagnosed as such by the app, with the other one having a score in the uncertain range. Two patients were diagnosed as non-epilepsy by the two examiners: one was diagnosed correctly by the app and the other incorrectly as epilepsy. Therefore, the app had a sensitivity of 97%, and a specificity of 97% for the diagnosis of epileptic episodes, with a misdiagnosis rate of 6%.

### Assessment of provoked seizures

Five patients were thought to have acute symptomatic seizures by the app, but FF assessment considered that they all had recent-onset epilepsy.

### Assessment of associated non-convulsive seizures

Information was available from 23 patients. FF examination identified five as having non-convulsive seizures. The app picked all of them but also identified 12 false positives, for whom FF assessment did not identify non-convulsive seizures. So, the sensitivity of the app compared to FF assessment was 100% and the specificity was 48% because of the false positives. The seizure types of the 12 false positives are shown in Table 4.

**Table 4:** Seizure types identified by the app but not by face-to-face assessment

Seizure type	Number
Focal, preserved awareness + focal, impaired awareness	6
Focal, preserved awareness	2
Myoclonic	2
Focal, impaired awareness	1
Focal, impaired awareness + myoclonic	1

*Determination of epilepsy type.*

FF assessment revealed that 28 of 30 patients had focal epilepsy and the other two had uncertain epilepsy type. Of the two cases, one was classified as focal and one as generalised type by the app. In addition, the app designated five of the focal epilepsies as acute symptomatic seizures (Table 5).

**Table 5:** Epilepsy types assigned by app versus FF assessment

Face-to-Face		App	
Focal	28	Focal	22
		Acute symptomatic	5
		Generalised	1
Uncertain	2	Focal	1
		Generalised	1

The misdiagnosis by the app mainly occurred in diagnosing patients with new-onset focal epilepsy as acute symptomatic seizures. This problem was associated with the algorithm used by the app.

### Appropriateness of treatment recommendations

Advice from the app results suggested a low dose of carbamazepine for six patients who were untreated at presentation, with the dose to be subsequently increased or other drugs added if necessary. Retrospective review by the neurologists showed that the advice given to all the patients, except the one with misdiagnosis, was appropriate (97%). Overall, eight of the 31 patients with epilepsy were seizure-free within 3 months before FF presentation (27%). Twenty-two patients felt better (71%), seven felt the same (23%), and two worse (6%), one of whom was a lady who switched her antiepileptic drug carbamazepine to levetiracetam according to her obstetrician’s prescription due to concerns on the risk caused by the drug to the baby, which caused worsening of her seizures. The other was an 18-year-old man who admitted to have poor adherence to carbamazepine at the dose of 800 mg daily.

## Discussion

### Feasibility

The current management app functions as an intelligent, epilepsy-specific, medical record which produces a summary with a management plan sent to an experienced neurologist. For untreated patients, it suggests a management plan when an experienced doctor is not available. Our results showed that the system was feasible, sustainable – it continues to be used after 18 months, and in particular timely – 85% of referrals were completed within 24 h.

### Quality

According to the US Institute of Medicine’s recommendation, the quality of the app can be assessed in six domains: safety, timeliness, efficiency, effectiveness, patient-centredness, and equity [13].

### Safety

The most important issue here is the misdiagnosis of non-epileptic episodes as epileptic and vice versa. Our app misdiagnosed two patients with epilepsy, one had psychogenic non-epileptic seizures on FF assessment, and the other falling within the uncertain range. The current app had a misdiagnosis rate of 6%, and sensitivity and specificity both of 97%. But epilepsy diagnosis is always difficult and no system is perfect; even FF consultation has been reported to have a rate of misdiagnosis ranging between 5% and 30% [14].

The system mistook newly-diagnosed epilepsy for acute symptomatic seizures probably due to the wording of the questions. This can be modified by revising the wording.

The app was also over-sensitive in diagnosing non-convulsive seizures, picking up all those recognised at FF consultation, but also 12 who were judged not having them. This piece of history review is the most difficult part as it is important to distinguish events separate from the main episode from events associated with it. This is not always easy even during FF consultation in one’s native language. The presence of non-convulsive seizures may potentially lead to the recording of myoclonic seizures by the app but not by FF examination. The epilepsy type was diagnosed as generalised by the app and uncertain by FF. As treatment options for females with generalised and uncertain epilepsy types are the same, the identification of non-convulsive seizures in this case had no effect on the management suggested.

The variability in FF classification of epilepsy type has not been studied so well as episode type, but one study [15] has suggested a rate comparable to the 27% in the present study, which was mostly due to the overdiagnosis of acute symptomatic seizures already referred to.

The treatment advice given by the system was appropriate in all cases apart from the one with misdiagnosis. The app-generated advice in six untreated patients was also regarded as appropriate. Comorbidity can affect the choice of drug used, but this was not an issue in the patients we assessed.

### Timeliness

There was a 98% response in 24 h and, even in those requiring a follow-up email, 85% of the referrals were complete within 24 h. This is unlikely to happen by FF examination in any country, however high-income it is.

### Efficiency

It took 5 to 10 min for the neurologist to reply to a referral, considerably less than the time of FF consultation for a newly-referred patient. This time efficiency depends first on the neurologist’s trust in the referrer and second, on the app-generated record that is both succinct and relevant to epilepsy management.

### Effectiveness

Effectiveness was assessed with a single question, to which 70% of the included patients responded to feel better after the treatment recommended by the app.

### Patient-centredness

Patient-centredness was not studied formally in this study; however, it is clear that this app saved both the cost of travelling to obtain a specialist’s opinion and the cost of the opinion itself.

### Equity

This referral system is more equitable than FF examination on three counts. First, the information, not the patient, travels directly to the specialist, no matter how remote the patient is from the urban centres which specialists tend to inhabit. Second, as this system is likely to be provided at a lower (if any) cost, it will be more available to poorer patients. Third, during the coronavirus disease 2019 pandemic, conventional FF care is dislocated and this method can readily serve as an alternative.

### Study weaknesses

One of the weaknesses of this study is that only a single referrer and a single specialist were involved. But that is how epilepsy services to LMICs are likely to evolve in the absence of investment from governments or large non-governmental organisations. And there has been precious little evidence that such investment is increasing [1] since the declaration by the World Health Assembly in 2015 [6]. Another weakness was the attendance rate of 56% at FF examination which has been dealt with earlier.

### Generalisability

This system of asynchronous telemedicine using an app to generate a disease-specific summary is easily replicable but requires both an open-minded referrer and an open-minded specialist; the app simply provides a medical record which links them. Financially for the NPHW, access to such a system may generate further paying referrals; for the specialist the activity can be either voluntary or paid. But both parties have to want to do something to address the well-publicised problems of epilepsy in LMICs.

## Conclusions

The epilepsy management app completed by an NPHW in Pakistan and reported by a UK-based neurologist provides safe, timely and efficient service for people with epilepsy, which can be an adjunct to existing FF neurology services. This model of care can be replicated in many other LMICs but requires some effort to establish. Making that effort could improve the lives of many people with epilepsy in LMICs.

## Data Availability

Data is available on request.

## Abbreviations

EEG: Electroencephalogram
FF: Face-to-face
LMICs: Low-and middle-income countries
NPHW: Non-physician health worker
UK: United Kingdom of Great Britain and Northern Ireland
